# Treatment and Prevention of Cardiogenic Arterial Thromboembolism in the Cat: A Systematic Review

**DOI:** 10.3390/ani15243539

**Published:** 2025-12-08

**Authors:** Giulia Arcuri, Pietro Bresolin, Carlo Guglielmini

**Affiliations:** Department of Animal Medicine, Production & Health, University of Padova, Viale dell’Università 16, 35020 Legnaro, Italy; giulia.arcuri@phd.unipd.it (G.A.); pietro.bresolin.1@studenti.unipd.it (P.B.)

**Keywords:** feline, vascular disease, cardiac disease, antithrombotic treatment

## Abstract

Feline cardiogenic arterial thromboembolism (ATE) is a severe complication of cardiac disease in cats, often causing severe clinical signs and poor prognosis. Despite its importance, standardized guidelines for prevention and treatment are lacking. This systematic review evaluated available evidence on preventive, acute, and chronic management strategies for feline cardiogenic ATE. A comprehensive search using PubMed, Scopus and Web of Science was performed, following PRISMA 2020 guidelines. Peer-reviewed studies investigating therapeutic interventions for ATE were included. Risk of bias was assessed using the SYRCLE tool. Nineteen studies involving 909 cats were included. Preventive therapy with clopidogrel and rivaroxaban improved survival. Acute multimodal treatment combining anticoagulant and antiplatelet drugs improved survival compared to monotherapy. Thrombolytic therapy showed some efficacy but had frequent severe complications. Long-term management with clopidogrel and rivaroxaban achieved the longest survival and lowest recurrence, while acetylsalicylic acid provided inconsistent benefits and more adverse effects. Eleven of the nineteen (58%) studies had high risk of bias due to small sample size and heterogeneous protocols. Current evidence supports dual therapy, particularly clopidogrel with rivaroxaban or enoxaparin, as the most effective and well-tolerated approach for prevention and treatment. Larger, standardized prospective trials are urgently needed to strengthen the evidence.

## Simple Summary

Arterial thromboembolism secondary to cardiac disease is a sudden and life-threatening condition in cats. It occurs when a blood clot forms in the heart and travels through an artery, blocking blood flow, most often in the hind limbs. Affected cats usually experience sudden pain and paralysis. Although arterial thromboembolism is a well-known complication of feline heart disease, clear and standardized recommendations for its prevention and treatment are still lacking. This review summarized and evaluated published studies investigating different treatment approaches to identify the safest and most effective ones. Nineteen studies including more than 900 cats were analyzed. The combined use of antiplatelet and anticoagulant treatments appeared more beneficial than using a single medication, with better survival and fewer recurrence rates. Therapies aimed at dissolving existing clots sometimes showed benefits but frequently caused serious side effects. However, since many studies were small and inconsistent, larger and standardized clinical trials are needed to determine strategies for preventing and treating cardiogenic arterial thromboembolism in cats.

## 1. Introduction

Cardiogenic feline arterial thromboembolism (ATE) is a serious condition that occurs when a thrombus originating in the left atrium enters the bloodstream and lodges in a distal arterial segment. Usually this happens in the distal aorta at the level of its trifurcation into the iliac arteries, but several other vessels, including the renal, cerebral, mesenteric, or brachial arteries, can be affected [1,2,3]. Arterial thromboembolism is a debilitating syndrome with acute clinical manifestations, including poikilothermy, pulselessness, paralysis/paresis, and pain in the affected limbs [1,4,5,6,7]. This condition most often affects both hind limbs (70–75% of cases) but can also be unilateral or involve a front limb [1,5,8]. Definitive diagnosis can be achieved by ultrasonographic assessment, either by directly visualizing the thrombus or by detecting an absence of Doppler signal in the affected artery. Furthermore, as a result of the ischemic event, an increased concentration of circulating lactate and glucose may be observed in the affected limbs, and their measurement, in comparison with that of the unaffected limbs, may provide supportive diagnostic value [1,9].

The major cause of cardiogenic ATE is feline cardiomyopathy [1,4], in particular hypertrophic cardiomyopathy (HCM), but also dilated (DCM), restrictive (RCM), and non-specific cardiomyopathy (NSCM) [6,8,10]. Other non-cardiac causes of ATE include neoplasia—usually pulmonary neoplasia—infection, inflammatory disease, hyperthyroidism, or iatrogenic causes (i.e., corticosteroids or progesterone administration) [1,8].

Identified risk factors for the development of cardiogenic ATE in cats include the presence of spontaneous echocardiographic contrast, left atrial enlargement, reduced left atrial contractility, impaired function of the left auricle, and a documented history of a previous ATE event [11,12,13]. As a result of left atrial dilation, cats with ATE often have concomitant congestive heart failure with pulmonary edema and/or pleural effusion. The prognosis for cats with ATE is generally poor, especially in cats with bilateral pelvic limb paralysis, compared to those with preserved motor function at admission or only one limb affected [5,8]. Lower rectal temperatures, higher lactate levels in affected limbs, and longer time to treatment are associated with worse outcomes [4,7,8].

The therapeutic approach in cats with ATE includes preventive, acute, and long-term management strategies. Preventive treatment is fundamental in cats with preclinical or clinical cardiomyopathy that are at risk of thromboembolic events. The most common treatment strategies include the use of antiplatelet or anticoagulant agents [11,14,15]. During an acute ATE event, it is critically important to stabilize the animal with pain control and oxygen supplementation and diuretics if CHF is present. Regarding specific treatment for ATE, a thromboprophylaxis therapy with antiplatelet and/or anticoagulant agents should be started [1,16,17,18,19]. In the long term, treatment focuses on managing the underlying cardiac disease and preventing recurrence of ATE [1,2,10,11].

Despite the clinical importance of feline ATE, standardized evidence-based treatment guidelines are not currently available. The lack of evidence on this topic, combined with the fact that ATE in cats can have a heterogeneous presentation, could increase the difficulty in establishing a standardized treatment approach.

Therefore, this study aims to provide a comprehensive, systematic, and critical evaluation of the existing literature about different treatment options used in cats with ATE, whether utilizing preventive, acute, or chronic approaches, in order to identify the best treatment protocols that ensure longer survival times and lower recurrence rates.

## 2. Materials and Methods

### 2.1. Stage 1—Searching Strategy

This systematic review has been registered in the International Prospective Register of Systematic Reviews (PROSPERO, registration number CRD42024595420). To conduct our systematic review, we adhered to the Preferred Reporting Items for Systematic Reviews and Meta-Analysis (PRISMA) 2020 guidelines [20] available at Supplementary Materials. Our search targeted full peer-reviewed studies addressing therapeutic approaches in cats with ATE secondary to heart disease. We systematically examined records of PubMed, Scopus, and Web of Science online databases from inception up to December 2024.

The search strategy involved the use of the following keywords and Boolean operators: “arterial OR aortic” AND thromboembolism AND “cat OR feline” AND “therapy OR treatment OR prevention”.

All identified records were catalogued using the online software Covidence (Covidence systematic review software, Veritas Health Innovation, Melbourne, Australia, https://www.covidence.org, accessed several times between 7 January and 3 March 2025), where the subsequent steps of screening, eligibility assessment, data extraction, and risk-of-bias assessment were performed. During this stage, duplicates were eliminated, either by software or manually.

### 2.2. Stage 2—Screening

For the initial screening, each retrieved record was independently assessed by two reviewers (GA and PB) based on the title and abstract. Studies were excluded if they were incomplete studies (i.e., if only the abstract was available), non-veterinary or non-clinical studies (including experimental induction of ATE in cats), review articles, and studies focused on species other than cats. Any disagreements between the initial reviewers were resolved by consulting with a third reviewer (CG).

### 2.3. Stage 3—Eligibility

After the initial screening, two authors (GA and PB) independently reviewed papers deemed eligible, examining the title, abstract, and full text. The same reviewers carefully assessed the full texts to determine whether they met the inclusion criteria. In case of discrepancies, a third author (CG) was consulted to reach a consensus.

The final corpus included only studies that satisfied the following criteria:

1. Peer-reviewed papers in the English language focusing on interventions for ATE secondary to heart disease in cats. Papers reporting interventions applied to any cat presenting or previously diagnosed with other forms of ATE (i.e., neoplastic or infectious ATE) were excluded.
2. Papers reporting primary research results, including case series, observational cohort studies, and randomized controlled trials (RCT). Studies such as literature reviews and single-case reports were excluded from the analysis.
3. Incomplete studies lacking essential data about treatments and their efficacy (i.e., missing data concerning the type and dosage of the active agent administered or regarding the effectiveness of treatment) were excluded.

### 2.4. Risk-of-Bias Assessment

To evaluate the quality of full-text articles deemed eligible, we employed the SYstematic Review Centre for Laboratory Animal Experimentation (SYRCLE)’s risk-of-bias tool for animal intervention studies [21].

The risk of bias was assessed independently by two reviewers (GA and PB), with any disagreement resolved through discussion with a third reviewer (CG).

The SYRCLE tool assesses six types of bias—selection bias, performance bias, detection bias, attrition bias, reporting bias, and other biases—through ten questions. Criteria are classified as low risk of bias when the answers to the questions are “yes,” high risk when the answers are “no,” or uncertain risk when there is uncertainty about the answer. The overall risk of bias was then assessed for each study based on the results obtained for each question.

### 2.5. Data Collection

For each article included in the systematic review, two independent reviewers (GA and PB) extracted the data. Specifically, for each study, we extracted data regarding the general characteristics of the study (year of publication, study design, and overall sample size), the characteristics of the population (number of cats with ATE, underlying heart disease, and other concomitant non-cardiac diseases), and the specific outcomes. In particular, data were collected for the therapeutic protocols used and their effects, such as improvement in clinical signs, median survival time (MST), percentage of recurrence of ATE, and time interval before its onset, as well as the percentage of adverse events. All these data were entered into a commercial spreadsheet and are presented in the following tables. For studies in which multiple treatment protocols were used, the results, when listed separately for each protocol, were reported in the respective columns of the table (i.e., improvement in clinical signs, number of dead cats, MST, occurrence/recurrence rate of ATE, and time interval for occurrence/recurrence of ATE), indicating the name of the drug used in that specific protocol. In the case of missing information, this was indicated in the tables as “not reported” (NR).

## 3. Results

### 3.1. Identification and Selection of Relevant Articles

**Figure 1:**
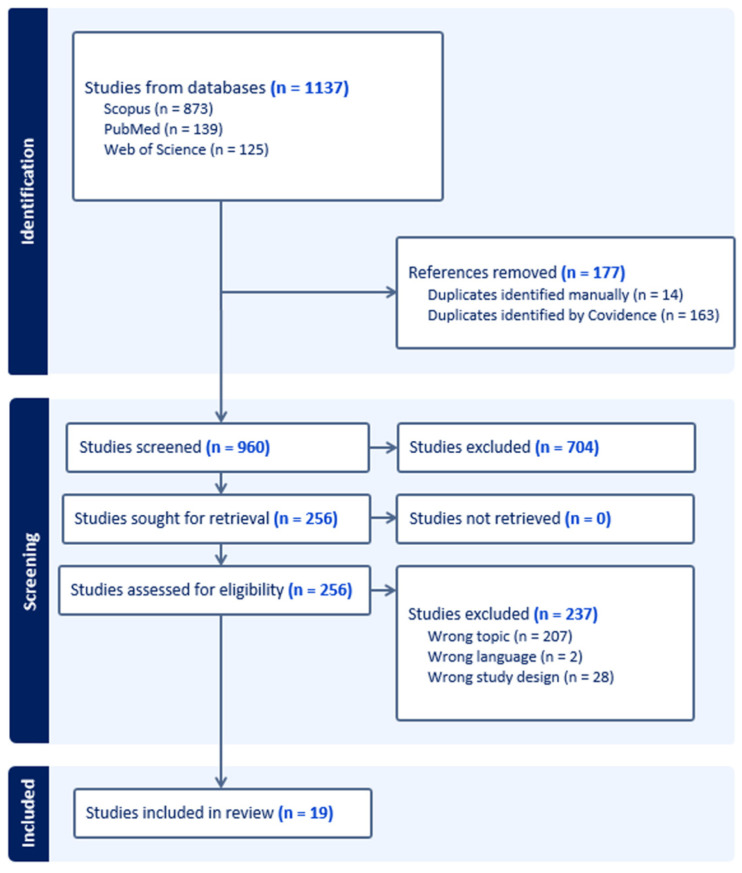
Flowchart of the literature search strategy.

A flow diagram of the search procedure is shown in Figure 1.

A total of 1137 records were initially identified from Scopus (*n* = 873), PubMed (*n* = 139), and Web of Science (*n* = 125). Exclusions were made for 177 records due to duplicate citations. Among the remaining 960 records, 704 were excluded during the first stage of screening for various reasons, namely, for being non-clinical veterinary studies, incomplete studies, review articles, or studies related to species other than cats. Following the assessment of 256 full-text articles for eligibility, 237 records were excluded due to being non-English studies (*n* = 2), being single-case reports (*n* = 28), or being studies related to cats with ATE secondary to non-cardiac disease or related to cats with cardiomyopathy but without ATE (*n* = 207). Consequently, our systematic review includes 19 studies involving 976 cats, of which 909 had ATE secondary to heart disease.

### 3.2. Study Characteristics and Risk-of-Bias Assessment

A comprehensive overview of study characteristics is presented in Table 1. Regarding study design, eleven studies (58%) were case series, six studies (32%) were RCT, and two studies were retrospective cohort studies and a prospective control trial (5% each). The extracted literature spanned a wide timespan, from 1999 to 2024, with the majority of articles (13/19 studies, 68%) published since 2014.

The most frequently reported underlying cardiac disease associated with ATE was HCM (16/19 studies, 84%), followed by RCM (8/19 studies, 42%), DCM (6/19 studies, 32%), and NSCM (5/19 studies, 26%). Other less-represented underlying cardiac diseases were atrial myopathy, atrial muscular dystrophy, mitral and tricuspid valve dysplasia, mitral or subaortic valve stenosis, and thyrotoxic cardiomyopathy (1/19 study, 5% each). In only one study (5%) the precise underlying cardiac disease was not reported.

**Figure 2:**
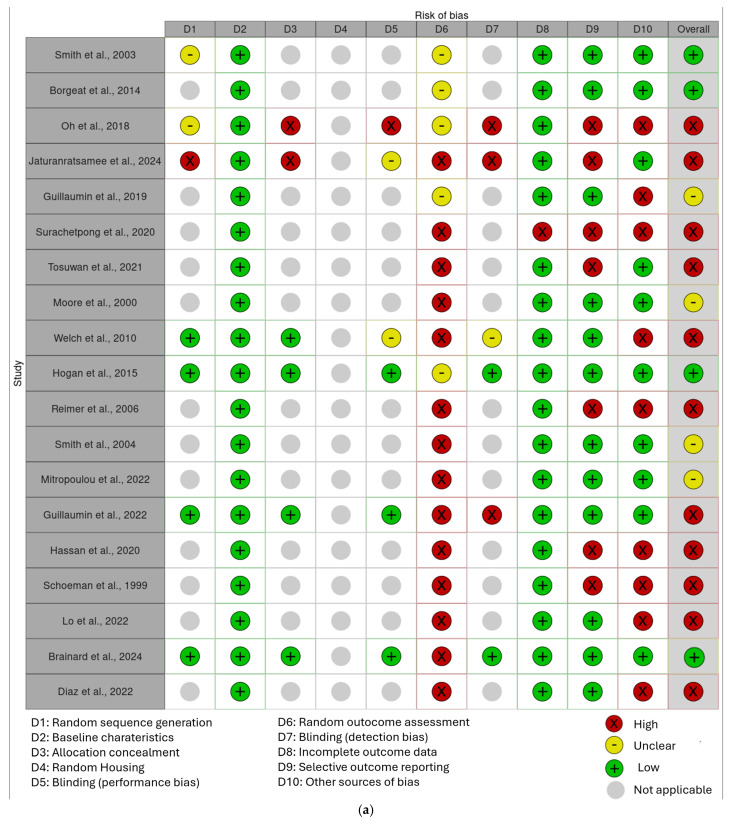

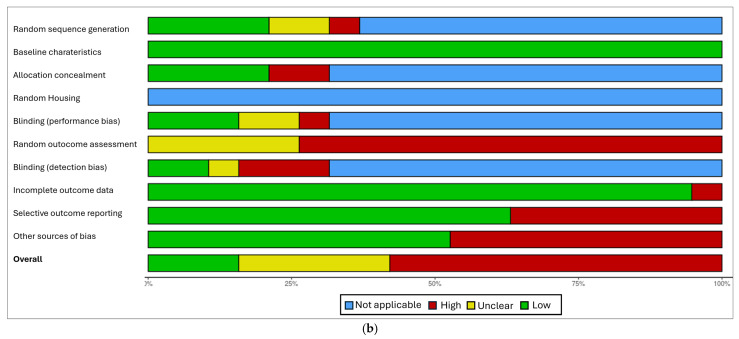
(**a**) “Traffic light” plots of the domain-level judgements for each result obtained with the Robvis tool [32]. (**b**) Weighted bar plots of the distribution of risk-of-bias judgements within each bias domain obtained with the Robvis tool [2,4,5,7,8,10,11,14,15,22,23,24,25,26,27,28,29,30,31,32].

The results of the risk of bias assessment are presented in Figure 2a,b.

In the final corpus, four studies (21%) resulted in a low risk of bias, four studies (21%) with a moderate risk of bias, and 11 studies (58%) with a high risk of bias, especially because of their study design (case series) or an insufficiently detailed reporting of results.

### 3.3. Treatment Approach

A comprehensive overview of the therapeutic strategies and their outcomes, including improvement in clinical signs, survival rates, MST, recurrence rate, and time to recurrence of ATE are shown in Table 2.

The most frequently reported treatments used for ATE secondary to heart disease in cats include both antiplatelet and anticoagulant drugs (14/19 studies, 74%). Thrombolytic therapy was described in eight studies (42%), while rheolytic therapy was mentioned only in one study (5%). Supportive care, including analgesics and cardiac medication, was also reported in fifteen studies (79%)

The strategies used for the preventive, acute, and chronic treatment of feline ATE and their results are described in more detail below.

#### 3.3.1. Preventive Treatment

Only three studies evaluated the efficacy of preventive treatment for the occurrence of cardiogenic ATE in cats (Table 3). The most effective protocol was the combined use of the antiplatelet agent clopidogrel and the anticoagulant rivaroxaban, with no reported ATE events (0/14 of treated cats) and an MST of 257 days. The use of dalteparin also showed a low incidence of ATE (1/23 of treated cats, 4%) with a shorter MST of 190 days. The incidence of treatment-related side effects was similar in both cases: 15% and 11%, respectively.

#### 3.3.2. Acute Treatment

Fifteen of nineteen studies reported the protocols used to manage acute ATE episodes, whose comprehensive overview is shown in Table 4. Among antiplatelet drugs, the most frequently used drug for the treatment of an ATE acute event was clopidogrel (7/15 studies, 47%), followed by acetylsalicylic acid (4/15 studies, 27%). Among anticoagulant therapies, unfractionated heparin and enoxaparin were both reported in six studies (40%), and rivaroxaban in one (7%). Among thrombolytic therapies, some studies reported on the use of tissue plasminogen activator (t-PA, 5/15 studies, 33%) and two studies on streptokinase or reolytic therapy (1/15 studies, 7% each).

In the studies included in this systematic review, the majority (8/15, 53%) investigated both the use of the specified drugs as monotherapy and in association, whereas only a few focused exclusively on either single-drug therapy (4/15, 26%) or combination therapy (3/15, 20%).

Not all studies reported outcomes separately for each drug combination. Good clinical improvement, defined as the return of femoral pulse and gradual recovery of motility, was observed as a result of the combined use of unfractionated heparin, enoxaparin, acetylsalicylic acid, and cerebrolisin (9/15 cats, 60%). Similar results were also obtained with the use of t-PA (up to 71% cats) and the combined use of enoxaparin and clopidogrel (up to 56% cats).

The highest survival rate (11/15 cats, 74%) was reported in one study that investigated the association between unfractionated heparin, enoxaparin, acetylsalicylic acid, and cerebrolisin. Among the other studies, the highest survival rates were observed in cats treated with enoxaparin and/or clopidogrel (up to 56% of cats survived), compared to those receiving unfractionated heparin and/or acetylsalicylic acid (up to 39% of cats survived). Good results were also obtained with t-PA (up to 60% of cats survived), but more side effects were found (up to 40%) than with enoxaparin and/or clopidogrel therapy (up to 22%). In a study with 11 cats treated with t-PA, all experienced side effects.

#### 3.3.3. Chronic Treatment

Seven studies investigated the effects of long-term treatment protocols on MST, recurrence rate of ATE, and time to relapse (Table 5). Of these, four studies (57%) used a monotherapy regimen, one study (14%) employed multitherapy, and two studies (28%) employed both. The best treatment, in terms of lower number of recurrences and longer survival time, was the combination of clopidogrel and rivaroxaban, with a recurrence rate of 17% (3/18 cats treated) and a MST up to 500 days.

The use of clopidogrel or rivaroxaban alone was associated with a slightly lower MST (up to 335 days and 296 days, respectively) and a higher recurrence rate compared with their combined use (up to 49% and 39%, respectively). On the contrary, in another study with a smaller population based on the use of only oral rivaroxaban, no recurrence was detected in all three cats treated.

A low molecular weight heparin, dalteparin, was also used, although it resulted in a slightly higher recurrence rate (25%, 5/20 cats) and a shorter MST (190 days). The use of acetylsalicylic acid alone resulted in a higher MST (402 days) with a 24% recurrence (4/17), as reported in one study. However, a more recent article on the use of acetylsalicylic acid alone at both high and low doses reported MST was much lower, 149 and 105 days, respectively, with a similar recurrence rate (11/44, 25%).

The longer time interval between the first ATE event and recurrence was observed with clopidogrel alone (663 days). Rivaroxaban alone also yielded a good result, with an interval of 513 days. Much more variable, on the other hand, was the outcome of the combination therapy of clopidogrel and rivaroxaban, with an interval ranging from 46 to 616 days. The most severe side effects were observed following a high-dose of acetylsalicylic acid (4/18 cats treated, 22%).

## 4. Discussion

Feline ATE represents one of the most severe complications that can occur in cats with heart disease. Despite its clinical relevance, standardized evidence-based guidelines for the treatment of ATE in cats are still lacking. Although a consensus (CURATIVE) on the use of antithrombotic therapy in veterinary medicine was published in 2019 [17,18,19] it does not clearly distinguish between primary prevention for cats at high risk of developing ATE, in-hospital treatment, and secondary prevention for ATE survivors.

The main reason for this gap is the limited high-quality evidence: most of the available data come from retrospective studies or small case series, often combining preventive, acute, and chronic management approaches in the same analysis, while RCTs are very uncommon. Indeed, among the studies included in this review, 13 were retrospective (12 case series and 1 cohort study), and only 6 were prospective studies. Furthermore, most studies were found to be at high risk of bias, for several reasons. First, reporting of treatment efficacy and adverse events was often incomplete, and pharmacological protocols varied widely within individual studies, either as monotherapy or combination therapy, but results were not always clearly stratified. Sample sizes were also highly variable: a total of 6 of the 19 studies included fewer than 20 cats, and only 2 studies enrolled more than 100 animals. Notably, the largest study (n = 250) did not clearly specify the underlying heart diseases of enrolled cats.

The present discussion focuses solely on the critical analysis of results related to preventive and therapeutic antithrombotic strategies, without addressing analgesic therapy or the management of underlying cardiomyopathies in cats with ATE.

### 4.1. Preventive Treatment

The preventive approach to the development of ATE in at-risk cats has been little investigated, as only three studies evaluated the efficacy of preventive treatment [11,14,15]. Of these, two were case series, while the other was a randomized study with a relatively small sample size (23 cats). All of these articles showed a moderate or high risk of bias. The most promising results were reported with the combined administration of the antiplatelet agent clopidogrel (18.75 mg/cat q24h PO) and rivaroxaban (2.5 mg/Kg q24h PO), an anticoagulant that binds directly to coagulation factor Xa [11]. In a case series on 14 treated cats, no ATE events occurred, and the MST reached 257 days. Similarly, treatment with the anticoagulant dalteparin (100 U/kg q12–24h SC), a low molecular weight heparin, also showed a low incidence of ATE (4%) in 23 treated cats, although the MST was shorter (190 days) [15]. The incidence of treatment-related side effects, mainly minor bleeding, was comparable between the two studies: 15% (5/32) and 11% (5/47), respectively. In another study, rivaroxaban was used for preventive treatment in 3 cats at risk of ATE for 60 days, and none of them developed an ATE event in that period. However, the article showed a high risk of bias, which, combined with the small sample size and the short period of administration of the drug, could limit the generalizability of these findings. [14]. Since only a few studies involving a small population have investigated preventive treatment in cats at risk of ATE, this topic has not yet been thoroughly explored and certainly requires future studies on a larger scale.

In humans, the presence of cardiovascular diseases such as atrial fibrillation (AF) or peripheral artery disease can predispose individuals to acute limb ischemia (ALI), a condition similar to feline ATE [33,34]. Previously, in patients with AF, prevention was generally achieved with coumarin anticoagulants such as warfarin, although the use of direct oral anticoagulants, such as rivaroxaban, has also been investigated, with good results [35]. Today, the combined use of arrhythmia-reducing ablation and left atrial appendage occlusion offers an interventional alternative for patients at risk [36]. In peripheral artery disease, therapy is generally based on antiplatelet agents such as acetylsalicylic acid, which may be combined with clopidogrel in high-risk cases [33,37]. In contrast, none of the articles included in this study used warfarin or acetylsalicylic acid for prophylactic purposes in cats at risk of ATE, given the high risk of severe hemorrhagic complications and the need for intensive monitoring. The aforementioned interventional strategies have not yet been implemented in veterinary medicine for cats with ATE, and investigating their feasibility, safety, and potential clinical benefit could represent a valuable and promising direction for future research.

### 4.2. Acute Treatment

The treatment of acute ATE has been the most extensively investigated aspect of feline ATE management, reported in 15 of the 19 studies included in this systematic review. However, the majority of them are case series (11/15), with relatively few RCTs available.

The best results in terms of survival outcomes have been achieved with multimodal strategies, in line with what has already been reported in the previous literature and in the CURATIVE consensus, in which the combined use of anticoagulants and antiplatelet agents is reported to be more effective than the use of anticoagulants alone [1,17,18,19,38]. In particular, the combination of clopidogrel (18.75 mg/cat q24h PO) and enoxaparin (1mg/kg q8–12h SC) has shown encouraging results [28,29,30], providing a higher MST and a better safety profile compared with acetylsalicylic acid and/or unfractionated heparin [8,22]. The latter agent, although widely used in human medicine [33,34], has frequently been associated with adverse effects in cats, including bleeding, gastrointestinal disorders, and renal complications [2,8]. It is important to note, however, that all studies investigating a multimodal approach were case series, most of them with a moderate to serious risk of bias, although sample sizes were generally larger than those in preventive studies. Furthermore, some of them simultaneously evaluated the approach to acute events with both monotherapy and multimodal therapy, but results were sometimes reported without clearly distinguishing between the protocol used, limiting the strength of the conclusions.

Recently, several studies [4,23,25,26,27,31] have investigated the use of thrombolytic therapy for acute ATE in cats. These include two small case series, three RCTs, and one retrospective cohort study, with the largest including 46 cats. The most widely used thrombolytic agent was t-PA, an enzyme that catalyzes the conversion of plasminogen to plasmin and plays a key role in endogenous thrombus dissolution. Although the CURATIVE study recognizes thrombolysis as a possible option in strictly selected cases [39], the guidelines of the American College of Veterinary Internal Medicine for the diagnosis and management of feline cardiomyopathies generally discourage its use due to the high risk of complications [16]. The use of t-PA (1mg/kg IV) in the studies included in our systematic review demonstrated good efficacy, with MSTs comparable to those obtained with multimodal antithrombotic therapy [4,25,27]. Nevertheless, severe adverse events, including hyperkalemia, acidosis, acute kidney injury, reperfusion injury, and sudden death, were common. One study reported adverse effects in all treated cats [25], underscoring the controversial nature of t-PA and supporting its use only in highly selected animals. Moreover, no significant survival difference was observed between thrombolytic therapy and conventional antithrombotic treatment in an RCT [4]. Streptokinase, a nonspecific plasminogen activator, was also evaluated in a retrospective study [23] in which 24% of treated cats (11/46) developed severe hemorrhagic complications. The limited number of RCTs and the small populations studied highlight the need for larger, prospective investigations to better assess the efficacy and safety of thrombolytic agents in feline ATE.

Given the high mortality and guarded prognosis associated with ATE, emerging therapies such as interventional and surgical thrombectomy are being investigated as potential alternatives to pharmacologic therapy in cats [1,24,38]. One prospective study [24] evaluated rheolytic thrombectomy in 6 cats, achieving successful dissolution of thrombus in 5 cases, but only 3 cats survived to discharge, with a similar survival rate to those treated only with medications. Importantly, ATE often occurs in cats with concurrent CHF and advanced cardiomyopathy, where the risks of anesthesia are considerable. Thus, medical management remains the mainstay of treatment. The feasibility of interventional approaches is further constrained by factors such as thrombus location, vessel diameter, limb viability, timing of intervention, and the patient’s overall stability.

In contrast, in humans, surgical and interventional strategies are considered essential to ensure rapid tissue reperfusion in ALI. Depending on the patient’s characteristics, comorbidities, and thrombus location, management may include surgical thrombectomy or endovascular approaches, such as catheter-directed thrombolysis, percutaneous thrombectomy, or stenting [33,34,40]. The most widely used active ingredient for thrombolysis is t-PA, with excellent outcomes, whereas streptokinase, as in cats, has been associated with lower efficacy and a higher risk of complications [33,41].

### 4.3. Chronic Treatment

Of the seven studies that investigated chronic antithrombotic treatment in cats with a history of ATE, four were case series [8,11,15,22] with a highly variable sample size, ranging from 32 to 127 animals, and three were RCTs [2,10,14] including from 23 to 75 cats. Similar to primary prevention of ATE, a multimodal approach has appeared to be the most effective strategy for chronic treatment. Specifically, the combination of the antiplatelet clopidogrel (18.75 mg/cat q24h PO) and the anticoagulant rivaroxaban (2.5mg/cat q24h PO) in a case series resulted in a lower recurrence rate and longer survival time [11] compared with clopidogrel [2,10] or rivaroxaban monotherapy [10], which were investigated in two RCT studies. The only exception was an RCT study that treated 3 cats with oral rivaroxaban alone, in which no recurrence was observed [14]. However, the very small sample size and the high risk of bias of this study limit the generalizability of this finding.

Unlike preventive therapy for a first ATE event, acetylsalicylic acid has been used in chronic treatment (5–81 mg/cat q72h PO) [2,8,22], but outcomes have been inconsistent, with a higher recurrence rate, variable survival time, as well as an increased incidence of adverse effects, especially following high-dose administration [8]. Overall, these results suggest poorer efficacy compared with the association of clopidogrel and rivaroxaban.

In humans, as with prophylactic therapy, chronic antithrombotic treatment depends on the underlying disease. Coumarin anticoagulants such as warfarin are preferred in patients with AF, whereas acetylsalicylic acid is commonly used for peripheral artery disease. In contrast to cats, acetylsalicylic acid is generally effective and well-tolerated in humans and may be combined with clopidogrel in high-risk cases [33,34,37]. No articles included in this systematic review investigated this therapeutic combination in cats.

Despite offering valuable insights, the present review is subject to several important limitations that must be acknowledged. First, the overall level of evidence across the studies included remains relatively low. Most data were derived from retrospective analyses or case series, with few RCTs available to date. The sample sizes themselves were another critical constraint. Most studies included relatively small populations with considerable heterogeneity in cardiac disease phenotype and clinical severity of ATE, with variable limb involvement. In terms of outcomes, the definitions and reporting approaches varied widely. Differences in how clinical improvement was defined, the inconsistent documentation regarding MST, the occurrence or recurrence rate of ATE, and the corresponding time interval before the onset make direct comparisons between studies challenging. Another important limitation lies in the fact that the protocols adopted across studies differed not only in drug choice but also on dosing and administration route variability, complicating both pharmacological evaluation and safety assessment. Lastly, the reporting of adverse effects was highly inconsistent. While some studies provided detailed accounts of side effects, others failed to mention them altogether. Furthermore, one study investigated treatment options for ATE not only in cats but also in dogs without always reporting separate results for the two species. Finally, the search strategy employed, which systematically applied the Boolean operators “AND” and “OR” to all document fields (title, abstract, and full text), was intended to ensure the most comprehensive retrieval of pertinent literature. Nevertheless, it must be acknowledged that the possibility of inadvertent omissions cannot be entirely ruled out, as factors such as the non-inclusion of certain documents within the consulted database may have limited the comprehensiveness of the search. This underreporting limits the reliable assessment of the overall efficacy and risk-benefit ratio of the protocols employed.

## 5. Conclusions

In conclusion, a dual therapy approach, most notably the combination of the antiplatelet agent clopidogrel and the anticoagulant rivaroxaban or enoxaparin, appears to be the most effective and well-tolerated strategy for the prevention and acute or chronic management of feline cardiogenic ATE. Thrombolytic agents like tPA, although potentially effective, are associated with high complication rates and should be used cautiously. However, it is important to consider that most of these results derive mainly from small case series and few randomized studies, with the majority of articles at risk of moderate or severe bias. The lack of standardized dosing regimens, heterogeneous study designs, and underreporting of adverse events highlight the need for larger prospective clinical trials.

## Figures and Tables

**Table 1:** Summary details of the 19 studies included in the final systematic review evaluating the therapeutic approach to feline arterial thromboembolism (ATE).

First Author (Reference)	Year	Study Design	Overall Sample Size	Cardiac Disease	ATE N (%)	Concomitant Non-CD N (%)
Schoeman, J.P. [22]	1999	CaS	44	HCM	44 (100)	NR
Moore, K.E. [23]	2000	CaS	46	HCM, DCM, MD/TD, AMD	46 (100)	2 (4.34)
Smith, S.A. [8]	2003	CaS	127	DCM, NSCM, HOCM, HCM, MS	127 (100)	18 (14)
Smith, C.E. [15]	2004	CaS	57	HCM, NSCM, RCM, DCM	20 (46,5)	4 (7)
Reimer, S.B. [24]	2006	PCT	6	HCM, RCM	6 (100)	NR
Welch, K.M. [25]	2010	RCT	11	NSCM	11 (100)	2 (18.18)
Borgeat, K. [5]	2014	CaS	250	NR	250 (100)	17 (6.8)
Hogan, D.F. [2]	2015	RCT	75	HCM, NSCM, HOCM, RCM, DCM, MS	75 (100)	NR
Oh, Y.I. [26]	2018	RCT	13	HCM, DCM	12 (92)	5
Guillaumin, J. [27]	2019	RCS	16	HCM, RCM, NSCM, CM	16 (100)	2 (12.5)
Surachetpong, S.D. [28]	2020	CaS	89	HCM, HOCM, NSCM, RCM, TCM	89 (100)	1 (1.4)
Hassan, M.H. [7]	2020	CaS	15	HCM	15 (100)	0 (0)
Tosuwan, J. [29]	2021	CaS	47	HCM, RCM	47 (100)	3 (6.3)
Mitropoulou, A. [30]	2022	CaS	36	HCM, HOCM, NSCM, AS, AM	36 (100)	3 (8)
Guillaumin, J. [4]	2022	RCT	40	NSCM	40 (100)	3 (7.5)
Lo, S.T. [11]	2022	CaS	32	HCM, RCM, SAS, CHD	18 (56.3)	NR
Diaz, D.M. [31]	2022	CaS	4	HCM, NSCM	4 (100)	3 (75)
Jaturanratsamee, K. [14]	2024	RCT	23	HCM	8 (34.8)	0 (0)
Brainard, B.M. [10]	2024	RCT	45	HCM, HOCM, NSCM, RCM, DCM	45 (100)	NR

AM: atrial myopathy; AMD: atrial muscular dystrophy; AS: atrial standstill; CaS: case series; CD: cardiac disease; CHD: congenital heart disease; CM: cardiac mass; DCM: dilated cardiomyopathy; HCM: hypertrophic cardiomyopathy; HOCM: hypertrophic obstructive cardiomyopathy; MD: mitral valve dysplasia; MS: mitral valve stenosis; N: number of cats; NSCM: nonspecific cardiomyopathy; NR: not reported; PCT: prospective controlled trial; RCM: restrictive cardiomyopathy; RCS: retrospective cohort study; RCT: randomized controlled trial; SAS: subaortic stenosis; TCM: thyrotoxic cardiomyopathy; TD: tricuspid valve dysplasia.

**Table 2:** Summary details of therapeutic approaches in cats with arterial thromboembolism (ATE) secondary to heart disease described in the 19 studies included in the final systematic review.

First Author (Reference)	Year	Treatment	Improvement in Clinical Sign N (%)	Death N (%)	MST Days (Range)	Recurrence of ATE N (%)	Time Interval for Recurrence of ATE Days (Range)
Schoeman, J.P. [22]	1999	Analg, AntiPlat, AntiCoag, CardT	NR	27/44 (61.3)	402 (3–2190)	4/17 (24)	30–360
Moore, K.E. [23]	2000	AntiCoag, Thrombo	RoP: 25/46 (54.3) RoMF: 14/46 (30.4)	31/46 (67.3)	51 (2–690)	7/15 (47)	100 (7–690)
Smith, S.A. [8]	2003	Analg, AntiPlat, AntiCoag, CardT	44/127 (35)	118/127 (93)	117	11/44 (25)	191 ± 152
Smith, C.E. [15]	2004	AntiPlat, AntiCoag	NR	36/47 (76.5)	190 (3–1223)	5/20 (25)	4–487
Reimer, S.B. [24]	2006	Thrombo, Reo	3/6 (50)	3/6 (50)	0–730	1/3 (33)	120
Welch, K.M. [25]	2010	Analg, Thrombo, CardT	7/11 (63)	4/11 (36) ^a^ 8/11 (73)	110–547	1/11 (9)	NR
Borgeat, K. [5]	2014	Analg, AntiPlat, AntiCoag, CardT, OT	68/250 (27)	30/68 (44) ^a^ 6/30 (20) ^b^	94 (7–2614)	14/30 (46)	118 (7–2614)
Hogan, D.F. [2]	2015	AntiPlat, CardT	NR	Clo 7/39 (18) Asa: 6/36 (17)	Clo: 248 Asa: 128	Clo: 19/39 (49) Asa: 27/36 (75)	Clo: 146 (7–990) Asa: 83 (6–883)
Oh, Y.I. [26]	2018	Analg, Thrombo	Group A: 8/10 (20) Group B: 5/7 (72)	Group A: variable (0–50) ^c^ Group B: variable (39–45) ^c^	NR	NR	NR
Guillaumin, J. [27]	2019	Analg, AntiPlat, AntiCoag, Thrombo, CardT, OT	Tpa: 9/16 (56.3) SOC: 11/38 (28.9)	Tpa: 7/16 (43.8) ^a^ SOC: 23/38 (60.5) ^a^	Tpa: 148 (61–1366)	Tpa: 4/7 (57)	Tpa: 106 (34–483)
Surachetpong, S.D. [28]	2020	AntiPlat, AntiCoag, CardT	Enx + Clo: 14/27 (52) Clo: 4/10 (40)	Enx + Clo: 52/79 (66) Clo: 6/10 (60)	31 (3–59)	4/89 (4.5)	10
Hassan, M.H. [7]	2020	Analg, AntiPlat, AntiCoag, Thromb, CardT, OT	9/15 (60)	4/15 (26)	NR	0/9 (0)	NR
Tosuwan, J. [29]	2021	Analg, AntiPlat, AntiCoag, CardT	NR	17/39 (43.6) ^a^	7 (3–202)	NR	NR
Mitropoulou, A. [30]	2022	Analg, AntiPlat, AntiCoag, CardT	20/36 (56)	19/36 (53) ^a^	NR	NR	NR
Guillaumin, J. [4]	2022	Analg, AntiPlat, AntiCoag, Thromb, CardT	Tpa: 12/20 (60) ^a^ Placebo: 8/20 (40) ^a^	Tpa: 9/20 (45) ^a^ Placebo: 11/20 (55) ^a^ Tpa: 15/20 (77) ^b^ Placebo: 20/20 (100) ^b^	Tpa: 45 (3–649) Placebo: 59 (1–156)	NR	NR
Lo, S.T. [11]	2022	AntiPlat, AntiCoag, CardT	NR	24/32 (75)	All: 257 Cats with ATE: 502	3/18 (16.7)	46–616
Diaz, D.M. [31]	2022	Thromb	1/4 (25)	3/4 (75)	NR	1/4 (25)	18
Jaturanratsamee, K. [14]	2024	AntiPlat, AntiCoag	NR	8/23 (35)	NR	Rvx: 0/6 (0)	NR
Brainard, B.M. [10]	2024	AntiPlat, AntiCoag, OT	NR	Clo: 7/19 (37) Rvx: 11/26 (42.3)	Clo: 335 (150–515) Rvx: 296 (209–510)	Clo: 7/19 (37) Rvx: 10/26 (39)	Clo: 663 Rvx: 513

Analg: analgesia; AntiCoag: anticoagulant treatment; AntiPlat: antiplatelet treatment; Asa: acetylsalicylic acid; CardT: cardiac treatment; Clo: clopidogrel; Enx: enoxaparin; MST: median survival time; N: number of cats; NR: not reported; OT: other treatment; Reo: reolytic treatment; RoP: return of pulse; RoMF: return of motor function; Rvx: rivaroxaban; SOC: standard of care; Thromb: thrombolytic treatment; Tpa: tissue plasminogen activator. ^a^ Short-term survival (within 7 days). ^b^ Long-term survival (within 1 year). ^c^ Population of both dogs and cats; specific results not always available for cats.

**Table 3:** Overview of the therapeutic protocol applied to prevent the onset of thromboembolic events in cats at risk of cardiogenic arterial thromboembolism (ATE).

First Author (Reference)	Year	Treatment	Treatment Duration Days (Range)	MST Days (Range)	Treatment-Related Side Effects N (%)	Occurence of ATE N (%)	Time Interval for Occurrence of ATE Days
Smith, C.E. [15]	2004	Dlt: 98.8 (47–220) U/kg q12–24h SC Asa: 3.3 (0.2–5.8) mg/kg q48–84h PO	Dlt: 172 (3–1223)	190 (3–1233)	5/47 (11)	Dlt: 1/23 (4)	78
Lo, S.T. [11]	2022	Rvx: 2.5 mg/kg q24h PO Clo: 18.75 mg/cat q24h PO	NR	257	5/32 (15)	0/14 (0)	NR
Jaturanratsamee, K. [14]	2024	Enx: 1 mg/kg q24h SC Clo: 3 mg/kg q24h PO Rvx: 2.5 mg/kg q24h PO	60	NR	Rvx: 0/3 (0)	NR	NR

Asa: acetylsalicylic acid; Clo: clopidogrel; Dlt: dalteparin; Enx: enoxaparin; MST: median survival time; N: number of cats; NR: not reported; PO: oral route of administration; Rvx: rivaroxaban; SC: subcutaneous route of administration; U: units.

**Table 4:** Overview of therapeutic approaches for management of acute thromboembolic events.

First Author (Reference)	Year	Treatment	Improvement in Clinical Signs N (%)	Death N (%)	Duration of Hospitalization Days (Range)	Treatment Related Side Effects N (%)
Schoeman, J.P. [22]	1999	AN, CardT, SUP Asa: 75 mg/cat q72h PO Ufh: 50–200 IU/kg q6–8h	NR	27/44 (61.3)	NR	NR
Moore, K.E. [23]	2000	Sk: 47(18–158) U/kg 4 h IV Ufh: 50–232 U/kg q6h SC	RoP: 25/46 (54.3) RoMF: 14/46 (30.4)	31/46 (67.3)	NR	27/46 (58)
Smith, S.A. [8]	2003	SUP, AN, CardT, Asa, Ufh: 75–500 U/kg IV	44/127 (35)	83/127 (65)	2 (0–10)	NR
Reimer, S.B. [24]	2006	Rt, Sk (45,000 IU INS)	3/6 (50)	3/6 (50)	NR	3/3 (100)
Welch, K.M. [25]	2010	Tpa groupA: 5 mg/cat 4 h CRI Tpa groupB: 1 mg bolus + 2.5 mg over 30 min + 1.5 mg over 1 h	7/11 (63)	4/11 (36) ^a^ 8/11 (73)	NR	11/11 (100)
Borgeat, K. [5]	2014	AN, CardT, Ufh, Asa, Clo, Ufh + Asa, Ufh + Clo, Ufh + Asa + Clo	68/250 (27)	220/250 (88) ^a^	2 (0–7)	NR
Oh, Y.I. [26]	2018	AN, Tpa group A: 20 mg 4 h CRI IV ^c^ Tpa group B: bolus + 0.5 mg/kg/h IV ^c^	Group A: 8/10 (20) ^c^ Group B: 5/7 (71.4) ^c^	Group A: NR (0–50) Group B: NR (39–45)	NR	7/17 (41.7) ^c^
Guillaumin, J. [27]	2019	AN, CardT, OT, SUP, Asa, Clo, Enx, Ufh, Ndr Tpa: 1 mg/kg IV	Tpa group: 9/16 (56) SOC group: 11/38 (28.9)	Tpa group: 7/16 (43.8) ^a^ SOC group: 23/38 (60.5) ^a^	Tpa group: 2 (0–11) SOC group: 1.5 (0–7)	Tpa group: 6/16 (37.5) SOC group: 14/38 (36.8)
Surachetpong, S.D. [28]	2020	CardT, Clo alone Enx 2.64 ± 0.86 mg/kg q8–12h SC + Clo	Enx + Clo: 14/27 (52) Clo: 4/10 (40)	Enx + Clo: 52/79 (66) Clo: 6/10 (60)	6 (3–14)	Enx + Clo: 4/79 (5)
Hassan, M.H. [7]	2020	CardT, SUP, OT Ufh: 300 IU/kg q12h SC Enx: 1 mL/kg q12h SC Crl: 2 mg/kg q24h for 6 d IM Asa: 10 mg/kg q36h PO	9/15 (60)	4/15 (26)	NR	NR
Tosuwan, J. [29]	2021	AN, CardT, Clo: 18.75 mg/cat q24h PO Enx: 1 mg/kg q6–12h, SC	NR	17/39 (44) ^a^	6.3	NR
Mitropoulou, A. [30]	2022	Enx: 1 mg/kg IV + 3 mg/kg/day CRI IV Clo: 75 mg/cat + 18.75 mg/cat q24h PO	20/36 (56)	19/36 (53)	7 (4–20)	8/36 (22)
Guillaumin, J. [4]	2022	CardT, AN, SUP, Enx/Dlt: 1 mg/kg SC Tpa: 5.15 mg/cat (3.7–6.0) IV Clo: 17.5 mg/cat q24h PO	Tpa: 12/20 (60) ^a^ Placebo: 8/20 (40) ^a^	Tpa: 9/20 (45) ^a^ Placebo:11/20 (55) ^a^ Tpa: 15/20 (77) ^b^ Placebo: 20/20 (100) ^b^	NR	Tpa: NR (55) Placebo: NR (38)
Lo, S.T. [11]	2022	CardT, Rvx: 2.5 mg/cat q24h PO Clo: 18.75 mg/cat q24h PO	NR	24/32 (75)	NR	5/32 (15.6)
Diaz, D.M. [31]	2022	Tpa: 1 (0.6–1.4) mg/kg IV	1/4 (25)	3/4 (75)	NR	NR

AN: analgesia; Asa: acetylsalicylic acid; CardT: cardiac treatment; Clo: clopidogrel; CRI: constant rate infusion; Crl: cerebrolysin; Dlt: dalteparin; Enx: enoxaparin; IM: intramuscular route of administration; INS: instillation; IU: international units; IV: intravenous route of administration; N: number of cats; Ndr: nadroparin; NR: not reported; OT: other treatment; PO: oral route of administration; Rt: rheolytic thrombectomy; Rvx: rivaroxaban; SC: subcutaneous route of administration; Sk: streptokinase; SOC: standard of care; SUP: supportive care; Tpa: tissue plasminogen activator; Ufh: unfractionated heparin. ^a^ Short-term survival (within 7 days). ^b^ Long-term survival (within 1 year). ^c^ Population of both cats and dogs; specific results not always available for cats.

**Table 5:** Overview of long-term management strategies aimed at reducing recurrence rates of cardiogenic arterial thromboembolism (ATE) in cats.

First Author (Reference)	Year	Treatment	Survival Time Days (Range)	Recurrence of ATE N (%)	Time Interval for Recurrence of ATE Days (Range)	Treatment Related Side Effects N (%)
Schoeman, J.P. [22]	1999	Asa: 75 mg/cat q72h PO	402 (3–2190)	4/17 (24)	NR	NR
Smith, S.A. [8]	2003	Hda: 40 mg/cat q24–72h PO Lda: 5 mg/cat q72h PO	117 Hda:149 Lda: 105	11/44 (25)	191 ± 152	Hda: 4/18 (22) Lda: 1/24 (4)
Smith, C.E. [15]	2004	Dlt: 98.8 (47–220) U/kg q12–24h SC Asa: 3.3 (0.2–5.8) mg/kg q48–84h PO	190 (3–1223)	5/20 (25)	4–487	5/47 (11)
Hogan, D.F. [2]	2015	Clo: 18.75mg/cat q24h PO Asa: 81 mg tablet/cat q72h PO	Clo: 248 Asa: 128	Clo: 19/39 (49) Asa: 27/36 (75)	Clo: 146 (7–990) Asa: 83 (6–883)	Clo: 1/39 (2.5) Asa: 1/36 (2.7)
Lo, S.T. [11]	2022	Rvx: 2.5 mg/cat q24h PO Clo: 18.75 mg/cat q24h PO	Total: 257 Initial ATE event: 502	3/18 (16.7)	46–616	5/32 (16)
Jaturanratsamee, K. [14]	2024	Enx: 1 mg/kg q24h SC Clo: 3 mg/kg q24h PO Rvx: 2.5 mg/kg q24h PO	NR	Rvx: 0/3 (0)	NR	Rvx: 0/3 (0)
Brainard, B.M. [10]	2024	Rvx: 2.5 mg/cat q24h PO Clo: 18.75 mg/cat q24h PO	Clo: 335 (150–515) Rvx: 296 (209–510)	Clo: 7/19 (37) Rvx: 10/26 (39)	Clo: 663 Rvx: 513	Clo: 0/19 (0) Rvx: 2/26 (7.7)

ATE: arterial thromboembolism; Asa: acetylsalicylic acid; Clo: clopidogrel; Dlt: dalteparin; Enx: enoxaparin; Hda: high-dose acetylsalicylic acid; Lda: low-dose acetylsalicylic acid; N: number of cats; NR: not reported; PO: oral route of administration; Rvx: rivaroxaban; SC: subcutaneous route of administration; U: units.

## Data Availability

Newly generated data (reanalysed from the original work) are contained within the article.

## Supplementary Materials

The following supporting information can be downloaded at: https://www.mdpi.com/article/10.3390/ani15243539/s1. The PRISMA 2020 checklist used to guide the reporting of this systematic review. Reference [42] are cited in the supplementary materials.

## Abbreviations

The following abbreviations are used in this manuscript:

AF	Atrial Fibrillation
ALI	Acute Limb Ischemia
CHF	Congestive Heart Failure
CURATIVE	Consensus on the Rational Use of Antithrombotics and Thrombolytics in Veterinary Critical Care
DCM	Dilated Cardiomyopathy
HCM	Hypertrophic Cardiomyopathy
IV	Intravenous route of administration
MST	Median Survival Time
NSCM	Nonspecific Cardiomyopathy
PO	Oral route of administration
PRISMA	Preferred Reporting Items for Systematic Reviews and Meta-Analysis
PROSPERO	International Prospective Register of Systematic Reviews
RCM	Restrictive Cardiomyopathy
RCT	Randomized Controlled Trial
SC	Subcutaneous route of administration
SYRCLE	SYstematic Review Centre for Laboratory Animal Experimentation
t-PA	Tissue Plasminogen Activator
